# Influence of N-Substituents on the Adsorption Geometry of OH-Functionalized Chiral N-Heterocyclic Carbenes

**DOI:** 10.1021/acs.langmuir.1c01199

**Published:** 2021-08-09

**Authors:** Shahar Dery, Peter Bellotti, Tzipora Ben-Tzvi, Matthias Freitag, Tehila Shahar, Albano Cossaro, Alberto Verdini, Luca Floreano, Frank Glorius, Elad Gross

**Affiliations:** † Institute of Chemistry and The Center for Nanoscience and Nanotechnology, The Hebrew University, Jerusalem 91904, Israel; ‡ Organisch-Chemisches Institut, Westfälische Wilhelms-Universität Münster, Münster 48149, Germany; § CNR-IOM, Laboratorio Nazionale TASC, Basovizza SS-14, Trieste 34012, Italy

## Abstract

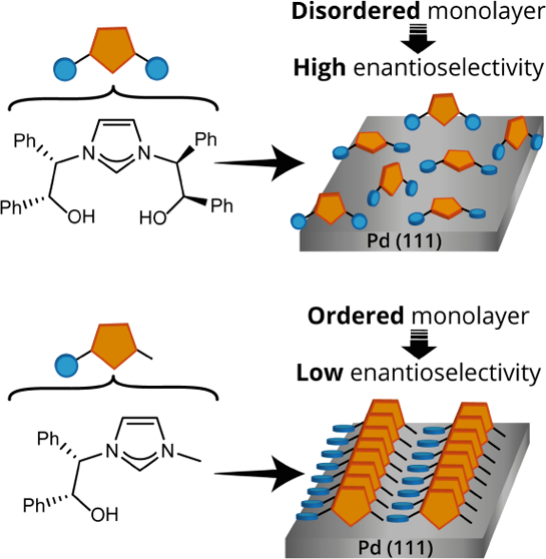

Adsorption of chiral molecules on heterogeneous catalysts is a simple approach for inducing an asymmetric environment to enable enantioselective reactivity. Although the concept of chiral induction is straightforward, its practical utilization is far from simple, and only a few examples toward the successful chiral induction by surface anchoring of asymmetric modifiers have been demonstrated so far. Elucidating the factors that lead to successful chiral induction is therefore a crucial step for understanding the mechanism by which chirality is transferred. Herein, we identify the adsorption geometry of OH-functionalized N-heterocyclic carbenes (NHCs), which are chemical analogues to chiral modifiers that successfully promoted α-arylation reactions once anchored on Pd nanoparticles. Polarized near-edge X-ray absorption fine structure (NEXAFS) measurements on Pd(111) revealed that NHCs that were associated with low enantioselectivity were characterized with a well-ordered structure, in which the imidazole ring was vertically positioned and the OH-functionalized side arms were flat-lying. OH-functionalized NHCs that were associated with high enantioselectivity revealed a disordered/flexible adsorption geometry, which potentially enabled better interaction between the OH group and the prochiral reactant.

## Introduction

The growing demand for enantiopure reagents in the pharmaceutical and fine chemicals industries led to the development of highly enantioselective homogeneous catalysts.^1−4^ However, a more sustainable approach requires a parallel development of recyclable enantioselective heterogeneous catalysts.^5−7^ In this context, chiral modification of catalytically active metal surfaces by adsorption of chiral molecules is a simple method for inducing chirality on a nonchiral surface, thus potentially generating enantioselective heterogeneous catalysts.^8−12^ Cinchona alkaloids are a prime example of highly efficient chiral modifiers, which following deposition on Pt or Pd surfaces catalyzed hydrogenation reactions with up to 98% enantiomeric excess (ee).^8,13−21^

Analysis of the molecular mechanism by which enantioselectivity is induced by cinchona alkaloids modifiers^8,10,11,18,22^ revealed that an efficient chiral modifier should consist of two core fragments: (a) an anchoring group that stabilizes the chiral modifier on the nonchiral surface, such as the quinolone ring in cinchona alkaloids, which induces π interactions with the underlying metal atoms, and (b) a chiral induction group that interacts with the prochiral reactant, for instance, via hydrogen bonding, to direct the adsorption geometry of the reactant on the catalytic surface.^23−25^ The enantiomeric induction efficiency in enantioselective heterogeneous catalysts is therefore controlled by the interplay between the anchoring group and the chiral induction group, which mediate the interaction between the catalytic surface and the prochiral reactant.

**Scheme 1:**
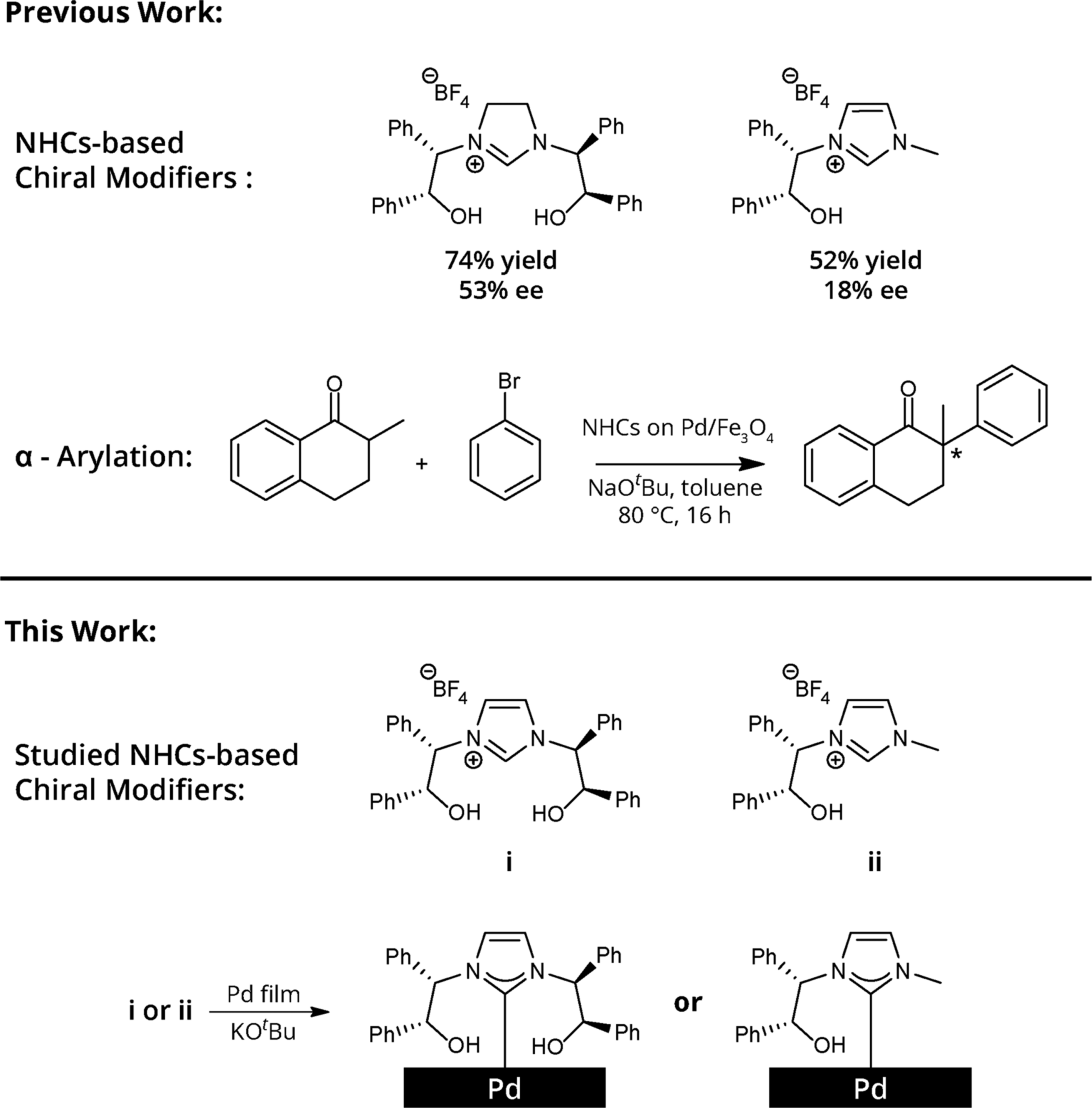
Previously Studied^42^ NHC-Based Chiral Modifiers and Their Effect on the Yield and Enantioselectivity of the Asymmetric α-Arylation of 2-Methyl-1-tetralone Using Bromobenzene (Top). In this work, the anchoring geometry of NHCs i and ii, which are chemical analogues to the NHC-based chiral modifiers, was elucidated by conducting polarized NEXAFS measurements (Bottom)

N-Heterocyclic carbenes (NHCs) represent a diversified class of donor ligands that are widely used for asymmetric induction in homogeneous catalysis.^26−28^ Unlike cinchona alkaloids, NHCs bind covalently to metallic surfaces and pursue high thermal stability along with wide chemical tunability of their N-substituents. The strong anchoring of NHCs on metal surfaces along with their versatile chemical functionality and controllable anchoring geometry^29−40^ makes them a highly promising surface ligand for chiral induction.^41^ The high potential of NHCs as chiral modifiers was demonstrated in the α-arylation reaction of 2-methyl-1-tetralone, which was effectively catalyzed following adsorption of OH-functionalized NHCs on Fe_3_O_4_-supported Pd nanoparticles (Scheme 1, top).^42^ NHC ligand that was decorated with 2-hydroxy-1,2-diphenylethyl chains on both N-substituents induced 53% ee (Scheme 1, top left), while NHC ligand that was decorated with a single 2-hydroxy-1,2-diphenylethyl N-substituent led to deteriorated enantioselectivity with 18% ee (Scheme 1, top right). It should be noted that the NHC ligands did not induce enantioselectivity enhancement in a homogeneous Pd catalyst,^42^ thus demonstrating that the steric hindrance of the chiral induction group is not sufficient by itself for enantiomeric induction.

To identify the variations in the anchoring geometry of these two ligands, which can be linked with their efficiency as chiral modifiers, we have synthesized two NHC chiral analogues and studied their adsorption geometry on well-defined Pd(111) by conducting polarized near-edge X-ray absorption fine structure (NEXAFS) measurements. The two NHC precursors featured identical imidazolium backbones and only differ in the identity of their N-substituents: **i** is functionalized with two 2-hydroxy-1,2-diphenylethyl arms, while **ii** is functionalized with a single 2-hydroxy-1,2-diphenylethyl arm and capped by a methyl at the other hand. By studying the differences in the adsorption geometry of these two NHCs, we were able to isolate the impact of N-substituents on the adsorption geometry. Differences in the surface orientation of **i** and **ii** were linked with variations in the enantioinduction efficiency of their chemical analogues.^42^ Chiral modifier **i**, an imidazolium analogue of the highly efficient NHC modifier that induced 53% ee, was characterized with a disordered adsorption geometry. The less-efficient chiral modifier **ii** (17% ee) showed an ordered geometry in which the imidazolyl backbone was vertically oriented and the phenyl groups were flat-lying on the Pd surface. These results suggest that the fixed and well-defined adsorption geometry of the chiral modifiers can be associated with deteriorated enantioselectivity while flexible and disordered geometry can induce high enantioselectivity.

## Experimental Section

### Pd(111) Cleaning Procedure

Pd(111) single crystals with a surface area of 0.5 cm^2^ (purchased from SPL) were cleaned under UHV conditions by three consecutive cycles of sputter (1 × 10^–6^ Torr Ar; 1.5 keV; 10 min) and annealing to 1000 K. The cleaning process was validated by C 1s X-ray photoelectron spectroscopy (XPS) measurements.

### Imidazolium Deprotonation and On-Surface NHC Deposition

Details about the synthesis of imidazolium salt precursors and NMR spectra are included in the Supporting Information. Free carbene was prepared by the addition of 0.3 g of KO^*t*^Bu to 8 mL of imidazolium salt solution, 0.18 g of (**i**) in tetrahydrofuran (THF), or 0.13 g of (**ii**) in dimethylformamide (DMF) and stirred at room temperature for 2 h. The Pd(111) single crystal was placed in a 20 mL scintillation vial in a glovebox. The freshly prepared carbene solution was transferred into the vial and covered the Pd surface. After 12 h, the single crystal was washed three times with 10 mL of THF or DMF. Finally, the Pd crystal was removed from the glovebox and washed with ethanol.

### Spectroscopic Measurements

X-ray absorption measurements were performed in partial electron yield using a Channeltron detector equipped with a front grid biased at a negative voltage (−230 V) to filter out low-energy secondary electrons. NEXAFS spectra at the carbon K-edge and the nitrogen K-edge were measured with the resolution set to ∼80 meV while keeping the sample at a constant grazing angle of 6°. The orientation of the surface with respect to the photon beam polarization was changed from s-polarization to close to p-polarization by rotating the sample coaxially to the photon beam axis. NEXAFS spectra were reported in the form of a normalized absorption amplitude (*I*/*I*_reference_), using NEXAFS measurement of a clean Pd(111) surface as a reference (*I*_reference_). X-ray photoelectron spectra of C 1s, N 1s, and Pd 3d were acquired. Binding energies were calibrated according to the Pd 3d_5/2_ position, located at 335.1 eV. Analysis of the XPS peaks and their fitting were performed by CasaXPS software.

## Results and Discussion

NHC-based chiral modifiers **i** and **ii** were synthesized by deprotonation of the corresponding imidazolium salt precursors and anchored on a Pd(111) single crystal (see the Supporting Information for experimental details).^34^ The adsorption geometry of NHCs on Pd(111) was analyzed by conducting X-ray photoelectron spectroscopy (XPS) and polarized NEXAFS measurements at ALOISA beamline of the Elettra synchrotron (Trieste, Italy).

**Figure 1:**
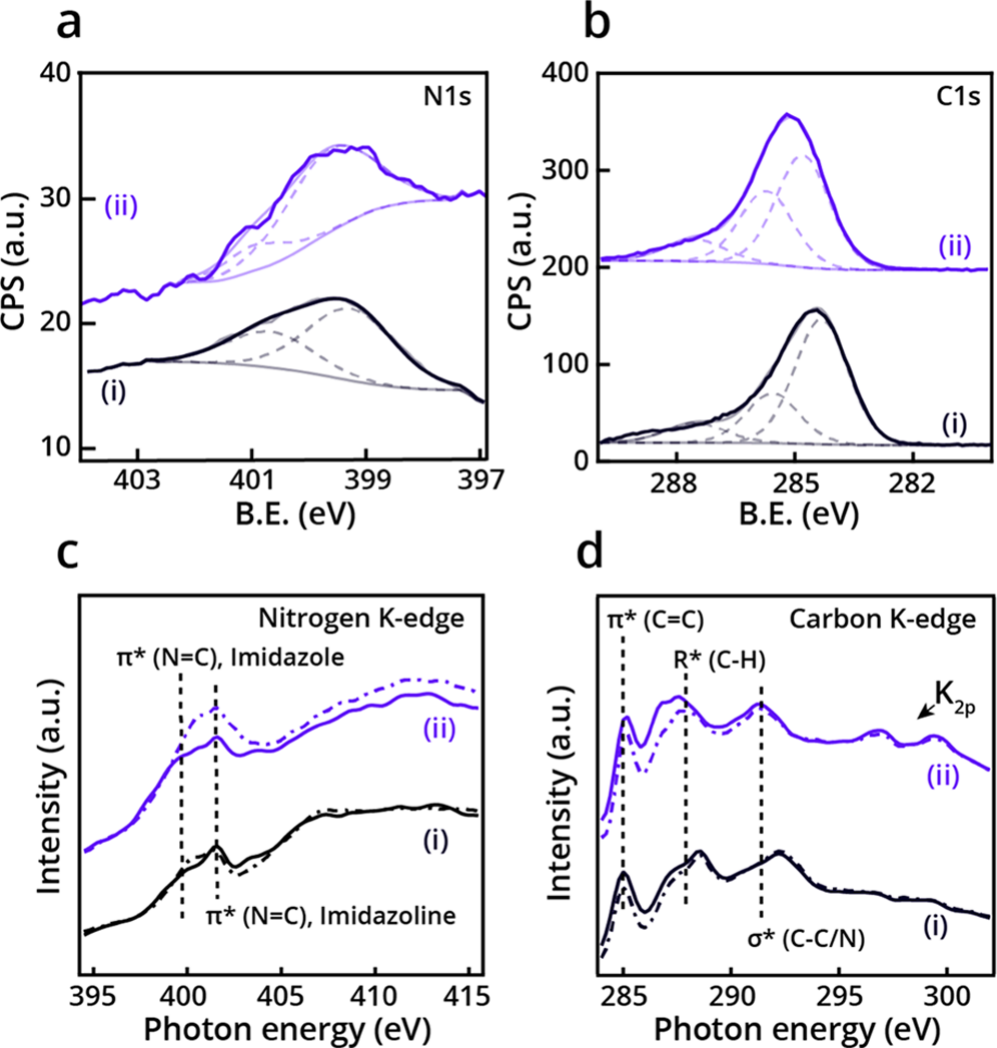
N 1s (a) and C 1s (b) XPS spectra of NHCs **i** and **ii** (black- and purple-colored spectra, respectively) on Pd(111). Gaussian fitting of the peaks was marked by dotted lines. Nitrogen (c) and carbon (d) K-edge NEXAFS spectra of NHCs **i** and **ii** (black- and purple-colored spectra, respectively). p- and s-polarized spectra were marked by solid and dotted lines, respectively.

Nitrogen signature in the N 1s XPS spectra indicated that the chiral modifiers were successfully adsorbed on the Pd(111) crystal (Figure 1a). The N 1s XPS signal of NHC **i** (Figure 1a, black-colored spectrum) exhibited a broad peak (397–402 eV) that was fitted by two Gaussians, centered at 399.5 and 400.8 eV and assigned to the C–N=C and N–C bonds of imidazole and imidazoline, respectively.^32^ N 1s XPS spectra of NHC **ii** revealed a similar pattern to that of NHC **i**. The presence of a small imidazoline signature can be correlated to a minor species in which the imidazole ring was hydrogenated following surface anchoring of the NHC.

The C 1s XPS signal of the two NHCs displayed a wide peak (283.4–288.5 eV) attributed to the presence of various carbon species on the surface (Figure 1b). The peak was fit by three Gaussians centered at 284.5, 285.6, and 287.6 eV and assigned to C=C, C–N/C–O, and C=O/COOH species, respectively.^43^ The detection of a C=O signature revealed that a fraction of the hydroxyl groups in both NHCs was oxidized to a carboxylic acid or aldehyde. Similar phenomena were previously observed in hydroxyl- and allyl-functionalized NHCs, in which the functional group was oxidized following surface anchoring of the NHC.^39,43^ The N 1s and C 1s XPS peak areas that were detected for NHCs **i** and **ii** were similar in their values, indicating that both ligands are characterized with similar surface density (Table S1).

Nitrogen and carbon K-edge NEXAFS measurements (Figure 1c,d, respectively) were performed to determine the adsorption geometry and chemical functionality of **i** and **ii** (marked by black- and purple-colored spectra, respectively) on Pd(111). The adsorption geometry of the two chiral modifiers was deduced by comparing the p- and s-polarized NEXAFS spectra (marked by solid and dotted lines, respectively, in Figure 1c,d). Nitrogen K-edge NEXAFS spectra of **i** (Figure 1c, black-colored spectra) displayed a dominant broad peak at 399–402 eV, assigned to the N 1s → π* transition of imidazole (399.5 eV) and imidazoline (401 eV).^32,43^ The comparable pattern of the p- and s-polarized spectra of NHC **i** (Figure 1c, black-colored spectra, solid and dotted lines, respectively) indicates that the monolayer is disordered with no preferred orientation of the imidazoline ring with respect to the Pd surface.

**Figure 2:**
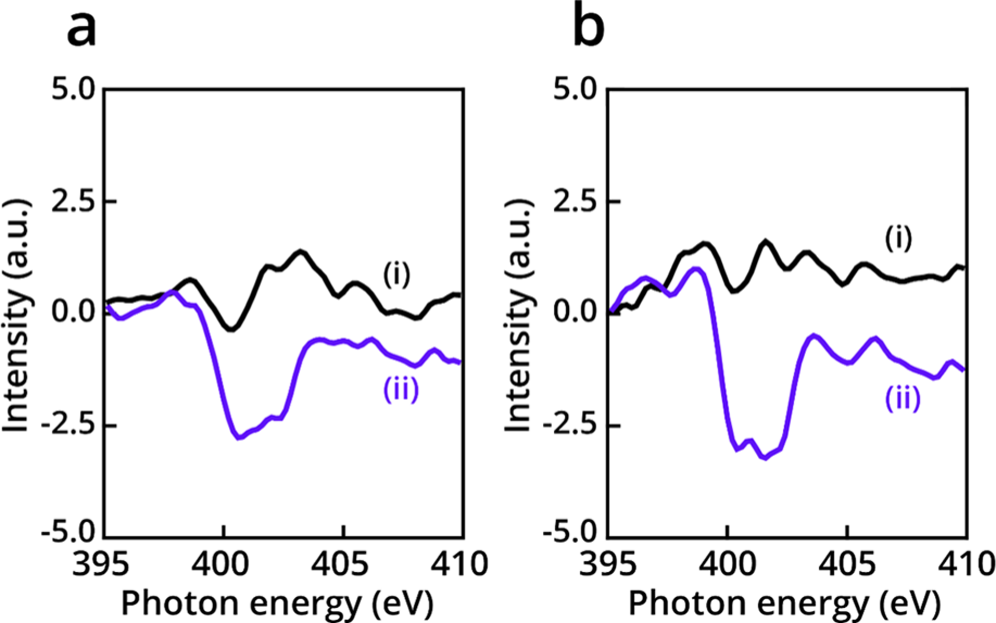
Linear dichroism analysis of nitrogen NEXAFS spectra of **i** and **ii** (black- and purple-colored spectra), respectively. Dichroism analysis was performed at rt (a) and after annealing to 80 °C (b).

Nitrogen K-edge NEXAFS spectra of NHC **ii** (Figure 1c, purple-colored spectra) showed a similar pattern to the spectra of NHC **i**. However, a higher amplitude was observed for the s-polarized than the p-polarized spectra of **ii** (Figure 1c, purple-colored spectra, solid and dotted lines, respectively). The negative linear dichroism (*I*_s_ > *I*_p_) that was detected for NHC **ii** indicates that the imidazole ring of NHC **ii** accumulated a more vertical orientation with respect to the Pd surface. Linear dichroism analysis (*I*_p_–*I*_s_) of nitrogen NEXAFS spectra (Figure 2a) displayed a featureless spectrum for NHC **i**, indicating a disordered orientation of the imidazole ring. In stark contrast, NHC **ii** showed a clear negative peak at 401 eV, indicating that the imidazole ring accumulated a more vertical orientation.

Carbon K-edge NEXAFS measurements revealed several peaks in the spectra of the two chiral NHCs (Figure 1d). A peak at 285.0 eV was assigned to the aromatic C 1s → π*_(C = N/C=C)_ transition. The peak at 287.2 eV was attributed to the R*_(C–H)_ transition, which is an indicative feature of C–H bonds.^32^ Additional two peaks were detected at 297–299 eV and attributed to potassium residues, which originated from the base that was used for deprotonation of the imidazolium salt precursors.^32^

For both NHCs **i** and **ii**, the amplitude of the p-polarized spectra (solid line) in the π* transition range was higher than that of the s-polarized spectra (dotted line), indicating a preference toward a flat-lying orientation of the phenyl rings. However, higher dichroism was identified for NHC **ii** (Figure 1d, purple-colored spectra), which indicates that the phenyl rings in **ii** have a higher tendency toward a flat-lying position.

Integration of the nitrogen and carbon NEXAFS data elucidated the differences in the adsorption geometry of the two ligands. NHC **ii** was characterized with an ordered structure, with the imidazole ring in a preferred upright position and the phenyl ring in a flat-lying position, while NHC **i** showed a lower affinity toward well-ordered adsorption geometry. The differences in the adsorption geometry of the two NHCs can explain the variation in the substituent efficacy of their chemical analogues.^42^

Lower reactivity and enantioselectivity were identified while using NHC **ii**, which displayed a vertical orientation of the imidazole ring and a flat-lying position of the phenyl rings. Higher reactivity and enantioselectivity were observed with NHC **i** analogue, which did not display a preferred surface orientation. It is likely that the disordered nature of NHC **i** enabled structural flexibility, which is essential for positioning the OH group in an optimal orientation to interact with the ketone group in the prochiral reactant 2-methyl-1-tetralone and direct its adsorption geometry on the catalytically active surface.^8,9^

**Figure 3:**
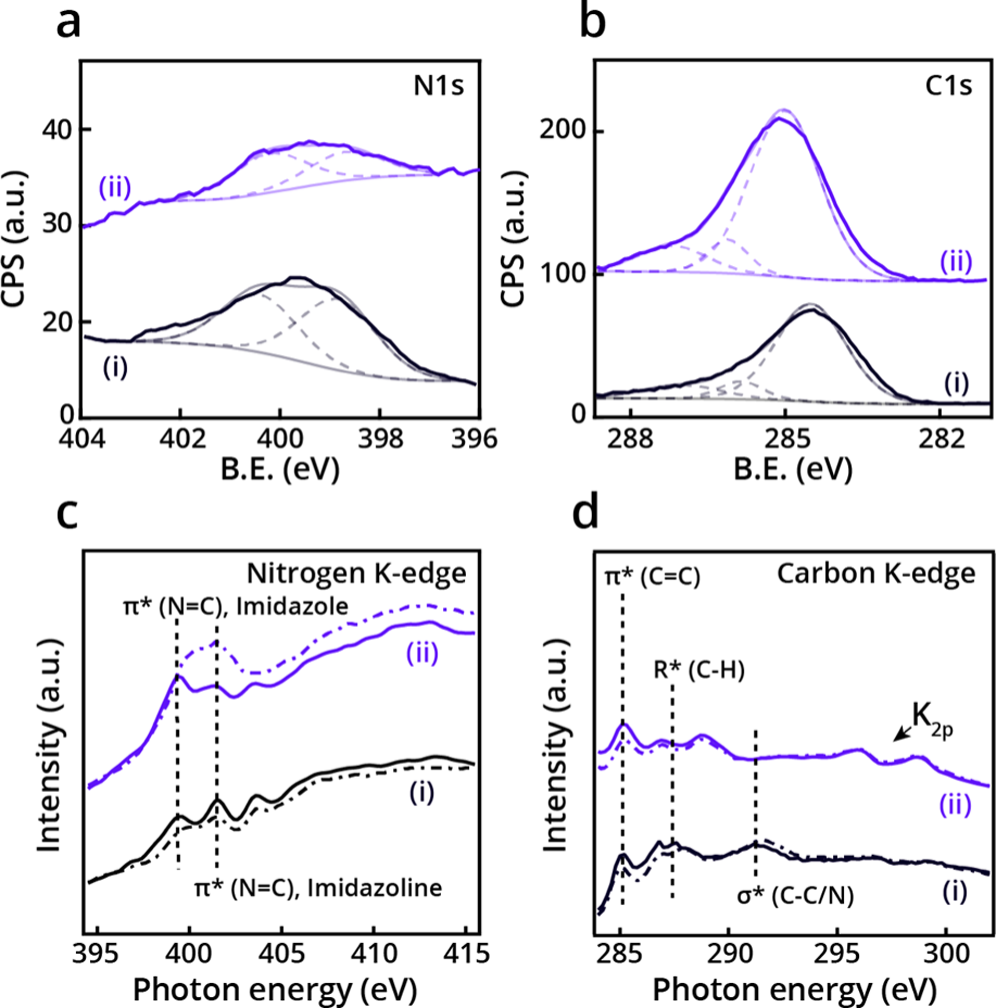
Spectroscopic data following annealing to 80 °C. N 1s (a) and C 1s (b) XPS spectra of NHCs **i** and **ii** (black- and purple-colored spectra, respectively) on Pd(111). Gaussian fitting of the peaks was marked by dotted lines. Nitrogen (c) and carbon (d) K-edge NEXAFS spectra of NHCs **i** and **ii** (black- and purple-colored spectra, respectively). p- and s-polarized spectra were marked by solid and dotted lines, respectively.

Pd(111) surfaces functionalized with NHCs **i** and **ii** were annealed to 80 °C, which represents the arylation reaction temperature, to identify the ways by which exposure to elevated temperature influences the adsorption geometry of NHCs. N 1s XPS spectra (Figure 3a,b, respectively) of the NHCs revealed that the ratio of the imidazole and imidazoline Gaussians (centered at 399.5 and 401.0 eV, respectively) became close to 1:1 after annealing, which indicates that the unsaturated bond was hydrogenated following annealing. Quantitative analysis of the XPS peak areas revealed that surface annealing led to a decrease in the C 1s and N 1s signals of NHC **ii**, correlated to partial decomposition or desorption of the surface-anchored ligand (Table S1).

Nitrogen NEXAFS spectra of NHC **i** following annealing (Figure 3c, black-colored spectra) revealed a new peak at 403.5 eV, which was correlated to oxidized nitrogen species^33^ that was induced by partial decomposition. Comparison of the p- and s-polarized nitrogen K-edge spectra showed higher intensity of the p-polarized spectrum, indicating that following annealing, the imidazole ring of **i** accumulated some preference toward a flat-lying orientation. Nitrogen NEXAFS spectra of NHC **ii** at 80 °C displayed an increase in the imidazole peak at 399.5 eV for the p-polarized spectrum (Figure 3c, dotted purple-colored spectrum) implying that dehydrogenation was facilitated by surface proximity.

Linear dichroism analysis of the nitrogen NEXAFS spectra at 80 °C further demonstrated the differences in the adsorption geometries of NHCs **i** and **ii** (Figure 2b). An increase in the negative dichroism of the imidazolyl fingerprint at 401.0 eV further proved the tilt toward a more vertical orientation of NHC **ii** backbone. Noticeably, the exposure to elevated temperatures has only increased the structural differences of the main ring between the two chiral modifiers. Thus, hydrogenation of the unsaturated bond in NHC **i** did not induce noticeable changes in its anchoring geometry. This result further establishes the connection between the adsorption geometry of NHC **i** and its saturated chemical analogue, which showed high efficiency as a chiral modifier.

The carbon NEXAFS spectra of NHC **i** displayed a negligible linear dichroism, indicating that the elevated temperature had only a minor influence on the adsorption geometry of the phenyl rings (Figure 3d, black colored spectra). Nonetheless, it should be noted that a decrease was observed in the amplitude of the peak at 285.0 eV, attributed to the C 1s → π*_(C=N/C=C)_ transition. This decrease further corroborates the quantitative XPS analysis, confirming partial decomposition of the phenyl rings. Carbon NEXAFS spectra of NHC **ii** showed also a decrease in the amplitude of the peak at 285.0 eV (Figure 3d, purple-colored spectra, p- and s-polarized spectrum in solid and dotted lines, respectively). Higher dichroism was detected in the carbon NEXAFS spectra of NHC **ii** than that of NHC **i** even after annealing to 80 °C.

Overall, the differences in the adsorption geometries of NHCs **i** and **ii** were more noticeable following annealing. NHC **i** mostly displayed a structural disorder under both temperatures, and this result was correlated with higher structural flexibility that enabled improved reactivity and enantioselectivity of its saturated chemical analogue. NHC **ii**, on the other hand, exhibited a well-ordered adsorption geometry characterized with the upright position of the imidazole ring and the flat-lying position of the phenyl rings. The structural order was further increased under elevated temperature. The fixed structure of NHC **ii** in which the OH-functionalized phenyl rings were positioned parallel to the surface was correlated with deteriorated reactivity and enantioselectivity.

The obtained results demonstrate that the two NHC ligands, which showed different efficiencies as chiral modifiers, differ in their adsorption geometry. The differences in the adsorption geometry will influence the ligands’ potential to induce steric hindrance and therefore can be linked with variations in their efficiency as chiral modifiers. It should be noted that exposure to reaction conditions involves the addition of various factors that can also affect the adsorption geometry of the ligands and their interaction with the prochiral reactant. It was demonstrated that the surface adsorption of hydrogen,^44,45^ solvent molecules,^46,47^ coadsorption of modifiers,^47,48^ and reactants^49−51^ can alter the reaction mechanism and the adsorption geometry of surface ligands. It cannot be excluded that similar factors will also modify the adsorption geometry of NHC ligands during the enantioselective α-arylation reaction. Therefore, complementary studies under reaction conditions will be beneficial for probing the adsorption geometry of NHC ligands at their active state and to determine the ways by which the adsorption geometry is influenced by exposure to the reaction environment.

## Conclusions

Dissimilarities in the adsorption geometry of two chiral NHC ligands, which differ in their N-substituents, were identified by conducting XPS and NEXAFS measurements. NHC **i**, which was linked with higher levels of enantioinduction (53% ee), displayed a disordered adsorption geometry on Pd(111). The disordered structure of NHC **i** enables high geometrical flexibility under reaction conditions, thus potentially leading to optimized interactions between the OH group and the ketone in the prochiral tetralone reactant. Conversely, NHC **ii**, which provided lower efficiency as a chiral modifier (18% ee), was characterized with a well-ordered structure in which the imidazolyl ring was vertically positioned and the phenyl rings were flat-lying on the surface. The well-ordered geometry of NHC **ii** likely limited its flexibility under reaction conditions and deteriorated its efficacy as a chiral modifier. The presented results therefore emphasize the importance of geometrical flexibility for optimizing the effectiveness of chiral induction by surface-anchored ligands on heterogeneous catalysts.

## Supporting Information Available

Ligand synthesis, experimental details, and XPS data analysis (PDF)
