# Supplementary material for: Influence of N-Substituents on the Adsorption Geometry of OH-Functionalized Chiral N-Heterocyclic Carbenes
Source: Langmuir. 2021 Aug 9;37(33):10029–35. doi: 10.1021/acs.langmuir.1c01199 (PMC9234974; doi:10.1021/acs.langmuir.1c01199)
Supplement: Supplementary file 1 — la1c01199_si_001.pdf [file la1c01199_si_001.pdf]

Supporting Information

The Influence of N-Substituents on the Adsorption Geometry of OH-functionalized Chiral N-Heterocyclic Carbenes

Shahar Dery[a], Peter Bellotti[b], Tzipora Ben-Tzvi[a], Matthias Freitag[b], Tehila Shahar[a], Albano Cossaro[c], Alberto Verdini[c], Luca Floreano[c], Frank Glorius[b]* and Elad Gross[a]*

[a] Institute of Chemistry and The Center for Nanoscience and Nanotechnology, The Hebrew University, Jerusalem 91904, Israel

[b] Organisch-Chemisches Institut, Westfälische Wilhelms-Universität Münster, Münster 48149, Germany

[c] CNR-IOM, Laboratorio Nazionale TASC, Basovizza SS-14, Trieste 34012, Italy

Experimental Section

Synthesis of the imidazolium salts. Unless otherwise stated, reactions were performed in oven-dried glassware under argon atmosphere. The reaction temperature refers to the measured value of the heating medium (oil bath, air or metal heating block). Chemicals were purchased from Aldrich, Alfa Aesar, Merck and ACROS and used with without further purification, unless otherwise stated. Reaction solvents were purchased from ACROS Organics (H_2O < 50 ppm), stored under molecular sieves and collected under positive argon pressure. Reactions were monitored by thin layer chromatography using pre-coated aluminum plates Merck silica gel $F60_{254}$ and visualized using 254 nm UV light or stained using basic $KMnO_4$ aqueous solutions (2 g $KMnO_4$, 10 g K_2CO_3, 0.3 g of NaOH in 200 ml of deionized water). Flash column chromatography was performed using technical grade solvent, purified by means of atmospheric pressure distillation. Silica gel (0.040-0.063 mm, Merck) was used as stationary phase under a slight positive argon pressure. ^{1}H NMR spectra were recorded using Bruker Avance 300 or 400 and Agilent DD2 600 MHz spectrometers. All spectral data were acquired at 295 K. Chemicals shifts (δ) are quoted in parts per million (ppm) against tetramethylsilane (TMS, δ = 0.00 ppm). The following residual solvent signals were used as references for ^{1}H spectra: DMSO-d_6: δ_H 2.50 ppm. Coupling constants (J) are reported in Hertz (Hz) to the nearest 0.1 Hz. The multiplicity abbreviations used (or combinations thereof) are: s = singlet, d = doublet, t = triplet, m = multiplet.

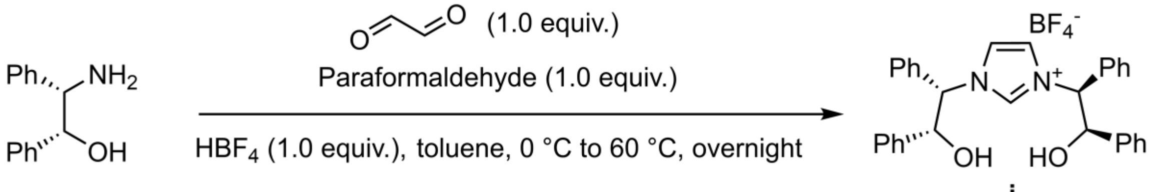

i

1,3-Bis((1*S*,2*R*)-2-hydroxy-1,2-diphenylethyl)-1*H*-imidazolium tetrafluoroborate (i). (1*S*,2*R*)-Amino-1,2-diphenylethanol (533 mg, 2.50 mmol, 2.0 equiv.) was added to a solution of paraformaldehyde (37.5 mg, 1.25 mmol, 1.0 equiv) and glyoxal (204 µL, 40 wt.% in H_2O, 1.25 mmol, 1.0 equiv.) in toluene (5 mL) at 0 °C. An aqueous solution of HBF_4 (144 µL, 50 wt.%, 1.25 mmol, 1.0 equiv.) was added dropwise over 5 min and the

solution was heated at 60 °C overnight. The solvent was removed *in vacuo* and the brownish solid residue was dissolved in DCM and dried over $MgSO_4$. The crude product was purified by flash column chromatography (DCM/MeOH = 95:5 to 80:20) to obtain the desired imidazolinium salt as an off-white solid (610 mg, 1.10 mmol, 88%). **R_f** (DCM/MeOH = 90:10): 0.56. **^{1}H NMR** (600 MHz, CD_2Cl_2) δ 9.29 (s, 1H), 7.38 – 7.29 (m, 2H), 7.29 – 7.16 (m, 20H), 6.95 (s, 4H), 5.70 (d, *J* = 4.4, 2H), 5.55 (d, *J* = 4.4, 2H). **^{13}C NMR** (150 MHz, CD_2Cl_2) δ 139.8, 135.8, 133.1, 129.8, 129.7, 129.0, 128.8, 128.5, 126.8, 122.2, 74.0, 70.0. **ATR-FTIR** (cm^{-1}): 3155, 2980, 1497, 1456, 1196, 1142, 1058, 827, 738, 699, 659. **HRMS** (ESI): m/z calculated for $[C_{31}H_{29}N_2O_2]^+$: 461.2224, found: 461.2223.

^{1}**H NMR** (600 MHz, CD_2Cl_2)

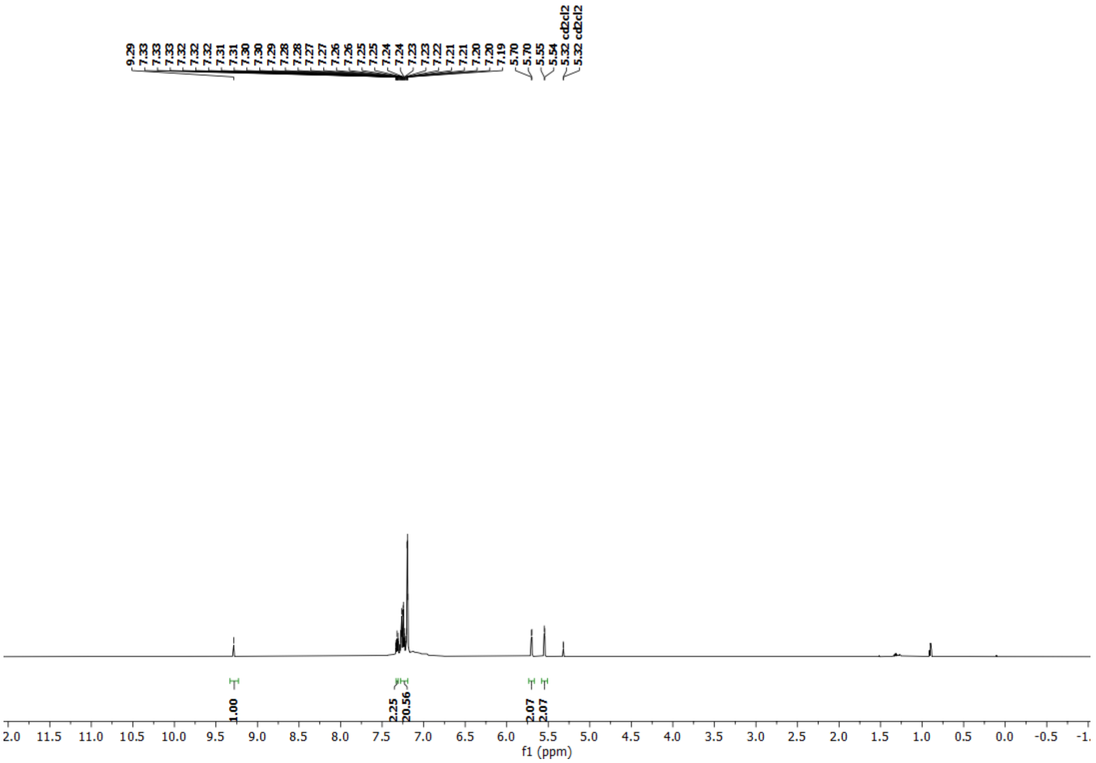

^{13}**C NMR** (150 MHz, CD_2Cl_2)

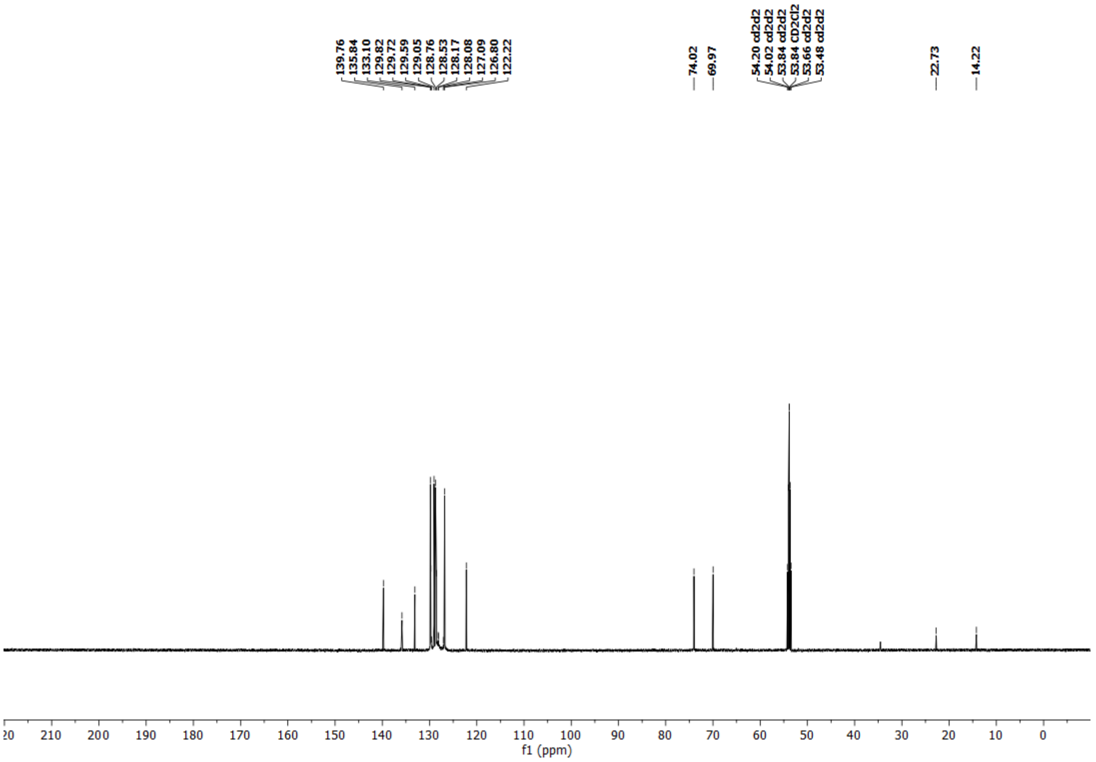

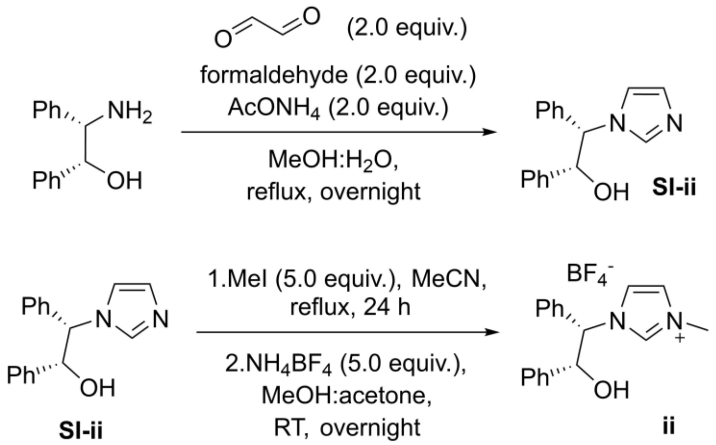

1-((1*S*,2*R*)-2-hydroxy-1,2-diphenylethyl)-3-methyl-1*H*-imidazol-3-ium tetrafluoroborate (ii).

The compound was synthesized according to the following two-step procedure.

Step 1. In a round-bottom flask equipped with a PTFE-coated stirring bar, glyoxal 40% *w/w* in water (2.28 ml, 20.0 mmol, 2.0 equiv.), formaldehyde 36% *w/w* in water (1.53 ml, 20.0 mmol, 2.0 equiv.), (1*R*,2*S*)-2-amino-1,2-diphenylethan-1-ol (2.13 g, 10.0 mmol, 1.0 equiv.) and ammonium acetate (1.54 g, 20.0 mmol, 2.0 equiv.) were charged, then MeOH (20 ml) was added. The reaction was vigorously stirred at reflux for 18 hours, then cooled to room temperature and the solvent was removed under reduced pressure. The residue was taken-up with KOH 2M (200 ml), then the resulting solution was extracted four times with CH_2Cl_2 (100 ml each time). The combined organic layers were dried over $MgSO_4$ and the solvent was removed under reduced pressure, affording the crude **SI-ii** as a light-red solid, which was used for the next step without further purification.

Step 2. In an oven-dried Schlenk tube equipped with a PTFE-coated stirring bar, the crude intermediate **SI-ii** (10.0 mmol from previous step), MeI (3.11 ml, 50.0 mmol, 5.0 equiv.) and dry MeCN (20 ml) were charged under argon and the reaction was heated at reflux for 24 hours, then cooled to room temperature. The solvent was removed under reduced pressure, then the residue was taken-up with MeOH:acetone 1:1 (40 ml), ammonium tetrafluoroborate (5.24 g, 50.0 mmol, 5.0 equiv.) was added and the corresponding

suspension was stirred at room temperature overnight. The reaction was concentrated under reduced pressure, then the crude residue was directly purified by flash column chromatography on silica (EtOAc:MeOH 4:1). The eluted fractions containing the product were concentrated *in vacuo* (orange solid) and the compound was further purified by three-fold trituration with *n*-pentane:EtOAc 1:1 to afford **ii** as a light yellow solid (2.73 g, 7.44 mmol, 74% over three steps).

^{1}H NMR (400 MHz, DMSO-d_6) δ 9.24 (s, 1H), 7.89 (t, J = 1.9 Hz, 1H), 7.63 – 7.51 (m, 3H), 7.40 – 7.21 (m, 8H), 6.23 (d, J = 4.7 Hz, 1H), 5.82 (d, J = 6.7 Hz, 1H), 5.49 (dd, J = 6.7, 4.6 Hz, 1H), 3.78 (s, 3H). The experimental data are in agreement with the literature report.[1]

[1] Ranganath, K. V.; Kloesges, J.; Schäfer, A. H.; Glorius, F. *Angew. Chem. Int. Ed.* **2010**, *49*, 7786.

Table S1. XPS peak area analysis of the NHCs-based monolayers

	**NHC i**		**NHC ii**	
Temperature	20 °C	80 °C	20 °C	80 °C
N1s/C1s	0.051	0.078	0.033	0.047
C1s/Pd3d	0.303	0.270	0.356	0.307
